# Comparative study of the effects of prenatal sevoflurane exposure at different cortical stages on forebrain development and maturation in offspring

**DOI:** 10.3389/fnins.2025.1556703

**Published:** 2025-04-03

**Authors:** Tianyuan Wang, Huandi Weng, Yalan Li

**Affiliations:** ^1^School of Pharmacy, Ningxia Medical University, Yinchuan, Ningxia, China; ^2^Department of Anesthesiology, The First Affiliated Hospital of Jinan University, Guangzhou, China; ^3^School of Pharmaceutical Sciences, Guangzhou University of Chinese Medicine, Guangzhou, China

**Keywords:** prenatal sevoflurane exposure, neuronal migration, embryonic cortical development stage, neurotoxicity, GABA

## Abstract

**Introduction:**

Brain development involves several critical stages, such as proliferation, neuronal migration, axonal pathfinding, and connection formation. Sevoflurane, a *γ*-aminobutyric acid (GABA) receptor agonist, is widely used as an inhaled general anesthetic. However, its impact on brain development has raised increasing concerns, particularly regarding prenatal exposure. This study aims to investigate the effects of prenatal sevoflurane exposure (PSE) at different cortical stages, focusing on its impact on the migration of glutamatergic and GABAergic neurons and neuronal behavior in offspring.

**Methods:**

PSE was administered at two critical prenatal stages: embryonic day (E) 12.5 and E18.5. Double *in situ* hybridization was used to identify the coexpression of GABA receptors in Pax6- and Mash1-positive cells in the forebrain. The radial migration of glutamatergic neurons and the tangential migration of GABAergic neurons were analyzed. Behavioral tests, including the open-field test, elevated plus-maze test, forced swim test, tail suspension test, sucrose preference test, and Morris water maze, were performed on offspring to assess anxiety-like behaviors, depression, and learning and memory impairments.

**Results:**

PSE inhibits the radial migration of glutamatergic neurons and promotes the tangential migration of GABAergic neurons. Specifically, early exposure (E12.5) inhibited the expression of the Pax6–Tbr2–Tbr1 cascade and the radial migration of Tbr1 in the ventral prefrontal cortex (PFC), whereas late exposure (E18.5) inhibited this process on the dorsal side. In addition, offspring mice with PSE exhibited increased anxiety-like behaviors, rather than depression, as demonstrated by reduced time spent in the center of the open-field test and in the open arms of the elevated plus-maze test. No significant differences were observed in the forced swim test, tail suspension test, or sucrose preference test. Furthermore, learning and memory impairments were observed in the Morris water maze.

**Conclusion:**

Our results indicate that PSE at E12.5 and E18.5 leads to abnormalities in the migration of glutamatergic and GABAergic neurons, affecting long-term anxiety-like behaviors and causing learning and memory impairments in offspring mice.

## Introduction

The safety of anesthetics in the developing brain has raised growing concerns (Ji et al., 2019). Approximately 0.15 to 2% of pregnant women undergo nonobstetric surgery every year (Van De Velde and De Buck, 2007). With the development of intrauterine fetal mirror surgery technology, this number continues to increase. Notably, pregnant women may use an adequate dose of volatile anesthetics to help maintain uterine quiescence and provide anesthesia to both the mother and fetus (Olutoye et al., 2020), but the impact and mechanism of exposure to volatile anesthetics during pregnancy on the fetus remain unclear.

Prenatal sevoflurane exposure (PSE) at key stages of brain development is reportedly related to long-term neural impairments in attention, cognition and emotion (Wang et al., 2018). The primary neurotoxic mechanisms of sevoflurane involve the activation of GABA receptors, the inhibition of NMDA receptors, and the involvement of multiple brain regions, targets, and various neurotransmitters (Makaryus et al., 2015). A recent cohort study revealed that prenatal surgery and anesthesia exposure result in children’s externalization behaviors (Ing et al., 2021). The mature brain contains glutamatergic excitatory and GABAergic inhibitory neurons, and their ratio remains relatively stable to maintain normal brain function. This highly depends on the processes of proliferation and migration of glutamatergic and GABAergic neurons during embryonic development (Bartolini et al., 2013), which are influenced by genetic and environmental factors. Many neurological and psychiatric abnormalities, such as schizophrenia, autism spectrum disorders and anxiety due to prefrontal cortex (PFC) dysfunction, are attributed to impaired brain development and maturation (Ferguson and Gao, 2018).

Cortical development begins with early neurogenesis, neuronal proliferation, migration and connection formation. In rodents, the initial stage of early neurogenesis occurs at embryonic day (E) 11.5 to E13.5 in the cerebral cortex (Gupta et al., 2002). Neural stem cells (NSCs) proliferate in the ventricular zone (VZ) and the subventricular region (SVZ), divide into neural progenitors in the intermediate zone (IZ) and then give rise to early glutamatergic neurons to migrate radially into different layers of the cortical wall (Lui et al., 2011). In contrast, GABAergic neurons are generated in the medial and lateral ganglionic eminence regions (MGE and LGE) of the ventral telencephalon and tangentially migrate into the cortex (Yamagishi et al., 2020). The migration peak occurs at which stages early axons reach their targets to form initial connections, and migrating neurons gradually occupy a specific destination to establish cortical lamination (Luján et al., 2005). At E18.5, the cerebral cortex enters the initial gliogenesis phase. While all excitatory neurons (spanning layers VI to II/III) have completed their migration and established a rudimentary layered organization—thereby forming the morphological framework of the six-layered structure (Di Bella et al., 2021).

GABA receptors include ionotropic GABA receptors (GABAAs), which are composed of 21 subunits (Luján et al., 2005), and metabotropic GABA receptors (GABABs), which are composed of 2 subunits (Billinton et al., 2001). Some subunits of GABA receptors are expressed predominantly during embryonic development, including the α2, α3, and α5 subunits of GABA_A_ receptors and the GABA_B1_ subunit of metabotropic GABA_B_ receptors (Laurie et al., 1992; Kim et al., 2003). During early cortical development, GABAergic neurons are excitatory and guide embryonic cortical neuron migration to appropriate positions after GABA_A_ receptors are activated (Luján et al., 2005; Tremblay et al., 2016). PSE has been reported to disrupt the tangential migration of GABAergic neurons in mouse embryos, impairing the structure and function of the inhibitory GABA circuit in the cerebral cortex after birth (Jiang et al., 2021). In late pregnancy, especially in the perinatal period, GABA_A_ receptor-mediated excitation disappears temporarily, and many developmental processes, such as neuronal proliferation, neuronal migration and synaptic formation, are not yet complete (Ben-Ari, 2014). This raises the possibility that PSE influences cortical development differently at different stages. However, whether PSE at different cortical development stages has a similar impact on brain development remains unknown. To address this question, we provided PSE at E12.5 and E18.5, the critical cortical development stages, and studied the immediate and persistent effects of PSE on neuronal proliferation and glutamatergic and GABAergic neuronal migration in offspring brains. The long-term effects on pyramidal neuron maturation and neurobehaviors were also investigated in offspring at the young adult stage.

## Materials and methods

### Animals and anesthesia

All procedures were performed in strict accordance with the recommendations in the Guide to the Care and Use of Experimental Animals. The experimental procedures were approved by the Experimental Animal Ethics Committee of Jinan University (Approval number: 20210830–18). C57BL/6 mice were purchased from Guangdong Experimental Animal Center. Inbred crossing started in the afternoon after adaptive feeding for 1 week. Vaginal plugs were checked the next morning, and positive results were noted as E0.5. Heterozygous GAD67-GFP knock-in mice were used to trace GABAergic neuronal migration. GAD67-GFP males were crossed with C57BL/6 females. The methods used to check the vaginal plugs and to provide PSE were the same as those described above. The animals were housed at 22 ± 1°C with 50–70% humidity and a 12-h light/dark cycle. Pregnant females were randomly divided into 3 groups: 2 PSE groups, including 4-h PSE groups at E12.5 and E18.5, and a control group without any treatment. In the PSE groups, pregnant mice were placed in a 40 × 40 × 25 cm chamber. Here, 2.5% sevoflurane (Baxter, s228L023) was delivered at a flow rate of 1 L/min for 4 h, and 50% oxygen was maintained. A thermostatically controlled heating pad was used to maintain the temperature inside the chamber, and the body temperature of each mouse was continuously monitored throughout the anesthesia process. The gas concentration was continuously monitored at the outlet of the test chamber (Bene View T8, Shenzhen Mindray Biomedical Electronics Co., Ltd., China).

### Double *in situ* hybridization (ISH)

E12.5 and E18.5 embryonic brains were prepared under RNAse-free conditions, and double ISH was performed using probes for mouse Pax6 (Cat. No. 412821), Mash1 (Cat. No. 313291), GABA_A-α3_ (Cat. No. 435021-C3) and GABA_B1_ (Cat. No. 501401-C2) designed by Advanced Cell Diagnostic, according to the protocol and using reagents provided in the RNAscope® Kit (Advanced Cell Diagnostics, Inc.). Briefly, PFA-fixed 14-μm frozen coronal sections were washed in 0.01 M PBS for 5 min and sequentially treated with RNAscope® hydrogen peroxide for 10 min at room temperature. RNAscope® target retrieval was performed for 30 min at 99–100°C. Then, samples were subject to RNAscope® Protease Plus for 30 min at 40°C, and hybridization with probes was performed for 2 h at 40°C in a HybEZ™ Humidity Control Tray (Advanced Cell Diagnostics, Inc.; Cat. No. 310012). The signal was revealed using an RNA Fluorescence Multichannel Staining Kit (Advanced Cell Diagnostics). Images were captured with a fluorescence microscope (Zeiss Imager A2, Germany).

### Immunofluorescence

For immunofluorescence staining, embryonic brains were fixed with 4% PFA at 4°C for 24 h, starting 2 h after sevoflurane exposure. Postnatal animals were perfused with 4% PFA, and their brains were postfixed with the same fixative overnight. Coronal sections (14 μm thick) were prepared using a cryostat (Leica, Germany). The sections were washed with 0.01 M phosphate-buffered saline (PBS) 3 times, incubated in 0.3% Triton-X (PBST) for 20 min, and blocked in 5% goat serum for 1 h. The sections were incubated with primary antibodies at 4°C overnight and washed three times (10 min each). The membranes were incubated with secondary antibody at room temperature for 1 h, washed three times (10 min each time) and stained with DAPI. The primary antibodies used for immunofluorescence included Pax6 (Abcam, ab195045), Tbr2 (Santa Cruz, sc69269), Tbr1 (Abcam, ab183032), PV (Millipore, mab1572), and GAD67 (Millipore, mab5406). The secondary antibodies used included donkey anti-rabbit 488 (Thermo Fisher, A21206), 594 (Thermo Fisher, A21207), donkey anti-mouse (488 Thermo Fisher, A21202), and donkey anti-sheep (647 Thermo Fisher, A32849). Each tissue block was repeated at least 3 times. Only representative images are displayed.

### Golgi staining

The Golgi staining experiment was performed according to the protocol of the FD Rapid GolgiStain™ Kit (PK401, FD NeuroTechnologies, Inc.). The whole fresh brain was quickly Golgi stained using an FD fast Golgi staining kit. The vibration slice thickness was 150 μm. Imaris software (Bitplane AG, Zurich, Switzerland) was used to select well-stained single pyramidal neurons in the superficial region of the mPFC (2/3) for three-dimensional reconstruction and dendritic analysis. To measure dendritic spine density, we used a 63× objective lens pair, and 10 μM segments of dendritic spines were counted. Twelve individual pyramidal neurons were obtained from each sample.

### Behavior tests

Each behavioral test was performed at a similar time interval on the day of testing at P23-30. One subgroup of mice was subjected to the open field test (OFT), elevated plus maze (EPM), tail suspension test (TST), forced swimming test (FST) and sucrose preference test (SPT), and the other subgroup of mice was subjected to the Morris water maze test (MWM).

### Open-field test (OFT)

The mice were placed gently in the center of a dimly lit open-field apparatus and allowed to move freely for 15 min. Their movements were recorded with a video camera and analyzed (Noldus EthoVision XT 7.1, Wageningen, Netherlands). The movement distance, speed and number of crossings in the central zone were calculated.

### Elevated plus maze (EPM)

The elevated plus maze (EPM) was composed of two open (56 * 10 * 1 cm) and two closed arms (56 * 10 * 40 cm) made of white Plexiglas. The maze was elevated 50 cm above the floor. After the open-field test, Day 1 mice were placed gently in the center of the cross of the open arm and closed arm and allowed to move freely for 5 min. Their movements were recorded with a video camera and analyzed using a Noldus EthoVision XT 7.1 (Wageningen, Netherlands). The time spent in the open arms was calculated.

### Tail suspension test (TST)

The mice were suspended for 6 min via the tail (2 cm from the end of the tail) using adhesive tape. The data were analyzed using Noldus EthoVision XT 7.1 to calculate the time spent in the immobile position during the last 4 min. If the velocity of the body center point was less than 1.5 cm/s, the animals were considered immobile.

### Forced swimming test (FST)

The mice were placed gently in a transparent Plexiglas cylinder (50 cm high and 15 cm diameter) filled with 25 cm deep water at 23 ± 1°C. Each animal was allowed to swim for 6 min. The data were analyzed using Noldus EthoVision XT 7.1 to calculate the duration of the immobile state during the last 4 min of the text. If the velocity of the body center point was less than 1.5 cm/s, the animals were considered immobile.

### Sucrose preference test (SPT)

All the animals were allowed access to tap water. They were then allowed access to one bottle of tap water and one bottle of 2% (wt./vol.) sucrose on the second day (Day 5). The positions of the bottles were switched on the third day (Day 6) to minimize egocentric orientation bias. On the 4th day (Day 7), we recorded the intake from the two bottles of water. Sucrose preference was quantified as the ratio of 2% sucrose intake weight to total liquid intake weight.

### Morris water maze (MWM)

The experiment used a water tank with a diameter of 120 cm and a height of 50 cm. The tank was filled with water to a height of 35 cm and was divided into four quadrants. There were fixed visual cues on the labyrinth wall (i.e., star, square, triangle and circle). The escape platform (10 * 10 cm^2^) was submerged in a quadrant 1.5 cm below the water surface. Swimming traces were recorded with a camera and analyzed using a computer system (Noldus EthoVision XT 7.1). The test scheme includes adaptive learning training at P23–P29, a position test and a detection test at P30.

### Statistical analysis

All the results are expressed as the means ± SEMs. GraphPad Prism 8.0.1 software was used for statistical comparison and plotting of the statistical data. According to the required sample size for statistical testing, all groups of experiments included data from at least three litters of animals (N: sample size of pregnant mice, n: sample size of offspring mice). For comparisons between two groups that followed a normal distribution, an unpaired two-tailed Student’s *t*-test was used. Continuous variables were tested for the assumption of normality and homogeneity of variance. The Kruskal–Wallis test, Welch’s *t*–test, and *t*–test were performed accordingly. Welch’s *t*–test was performed when homogeneity of variance was not met, and the Kruskal–Wallis test was performed if normality was not met. The Student’s *t*–test was performed only when both conditions were met. Mixed effect modelling was used to analyze repeatedly measured data. Differences with *p* < 0.05 were considered statistically significant. Multiple group comparisons were conducted using one-way ANOVA, two-way ANOVA, or Bonferroni *post hoc* correction. For the distribution of labelled positive cells in the PFC, the selected cortical coronal sections were uniformly divided into widths of 200 μm and 10 counting areas (bins). The results are expressed as the number of positive cells labelled in each counting area/area (number of cells/mm^2^). A *p* value<0.05 was considered statistically significant.

## Results

### GABA receptors are expressed in GABAergic and glutamatergic progenitors at different stages of cortical development

In the developing brain, the expansion and formation of the layered structure of the cerebral cortex depend on the migration of glutamatergic neurons and GABAergic neurons from different locations to different layers (Luján et al., 2005). Prior to migration, glutamatergic projection neurons and their progenitors express Pax6 (Hevner et al., 2006), whereas GABAergic interneurons express Mash1 (Letinic et al., 2002) in their germinal zones. We detected Pax6 and Mash1 mRNA expression with GABA_B1_ and GABA_A-α3_ receptors at E12.5 and E18.5 using double *in situ* hybridization in the control group.

**Figure 1:**
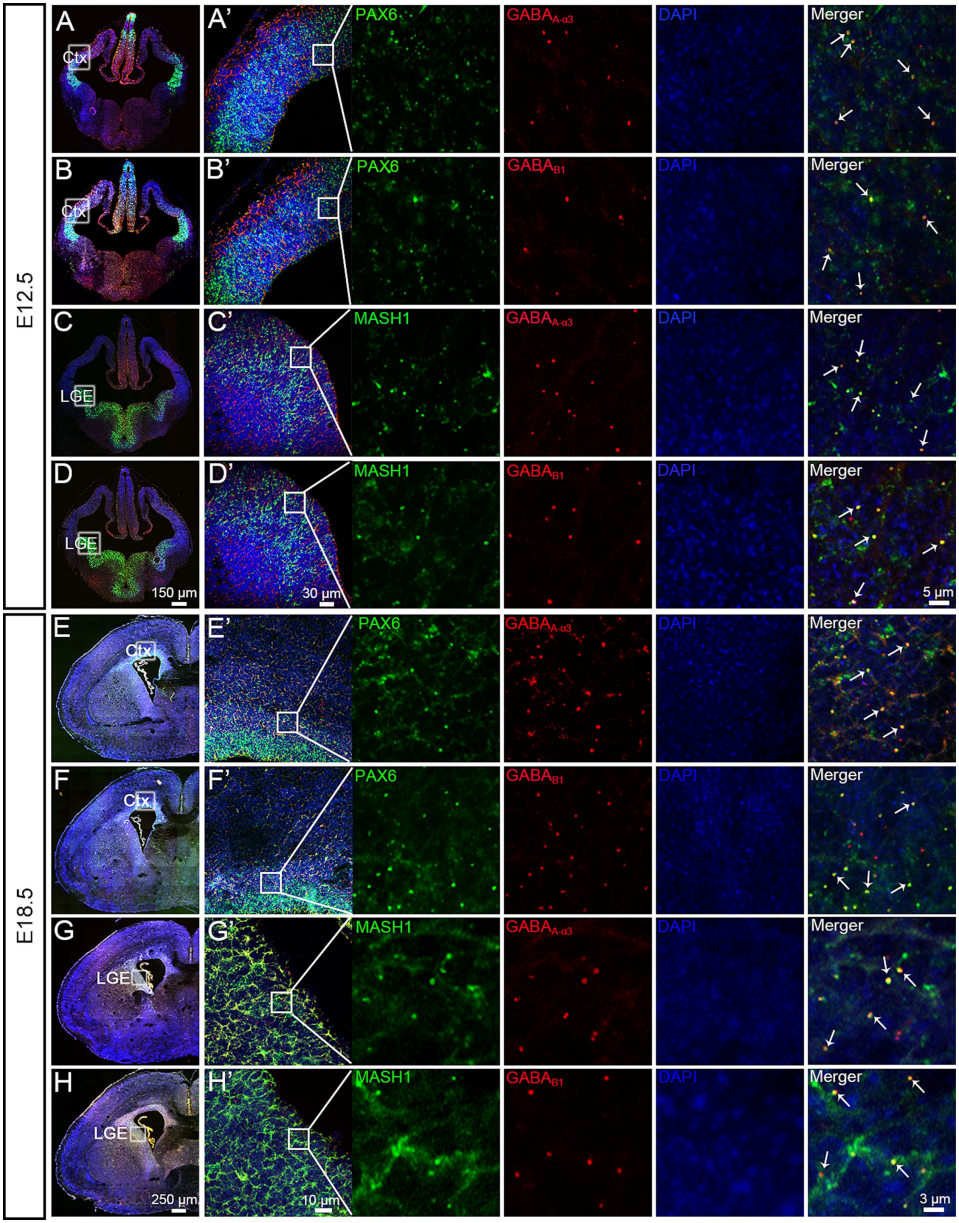
Pax6, Mash1, GABA_A-α3_ and GABA_B1_ mRNA expression. **(A–D)** At E12.5, Pax6, Mash1, GABA_A-α3_ and GABA_B1_ were subjected to *in situ* hybridization using RNAscope. Pax6 shows a strong signal in the VZ/SVZ of the CTX **(A,B)**. Mash1 shows a strong signal in the ventral telencephalon **(C,D)**. A partial enlargement of the image shows that GABA_B1_ and GABA_A-α3_ receptors colocalized with the expression of Pax6 and Mash1 (**A’–D’**, indicated by arrows). **(E–H)** At E18.5, Pax6, Mash1, GABA_A-α3_ and GABA_B1_ were subjected to in situ hybridization using RNAscope. Pax6 shows a strong signal in the VZ/SVZ of the CTX **(E,F)**. Mash1 shows a strong signal in the ventral telencephalon **(G,H)**. A partial enlargement of the image shows that GABA_B1_ and GABA_A-α3_ receptors colocalized with the expression of Pax6 and Mash1 (**E’–H’**, indicated by arrows). Ctx, cortex; LGE, lateral ganglionic eminence.

Pax6 is expressed in the VZ/SVZ of the PFC and colocalizes with the expression of GABA_B1_ and GABA_A-α3_ receptors (Figures 1A–B’,E–F’), Mash1 was expressed in the ganglionic eminence (GE) and colocalized with the expression of GABA_B1_ and GABA_A-α3_ receptors (Figures 1C–D’,G–H’) at two key cortical stages (E12.5 and E18.5). These results suggest that activating GABA receptors may affect the differentiation and migration of cortical excitatory and inhibitory neurons at different stages of cortical development.

### PSE at different cortical periods influences glutamatergic and GABAergic neuron migration and differentiation at E12.5 and E18.5

To assess the impact of PSE at different cortical stages on glutamatergic neuron migration, we prepared brain sections from the E12.5-sevo and E18.5-sevo groups and conducted immunofluorescence staining for Pax6 (radial glia), Tbr2 (intermediate precursor cells), and Tbr1 (postmitotic neurons). We also used GAD67-GFP knock-in mice (GAD67^+^/GFP) exposed to sevoflurane at E12.5 and E18.5 to observe the laminar distribution of GFP^+^ cells.

**Figure 2:**
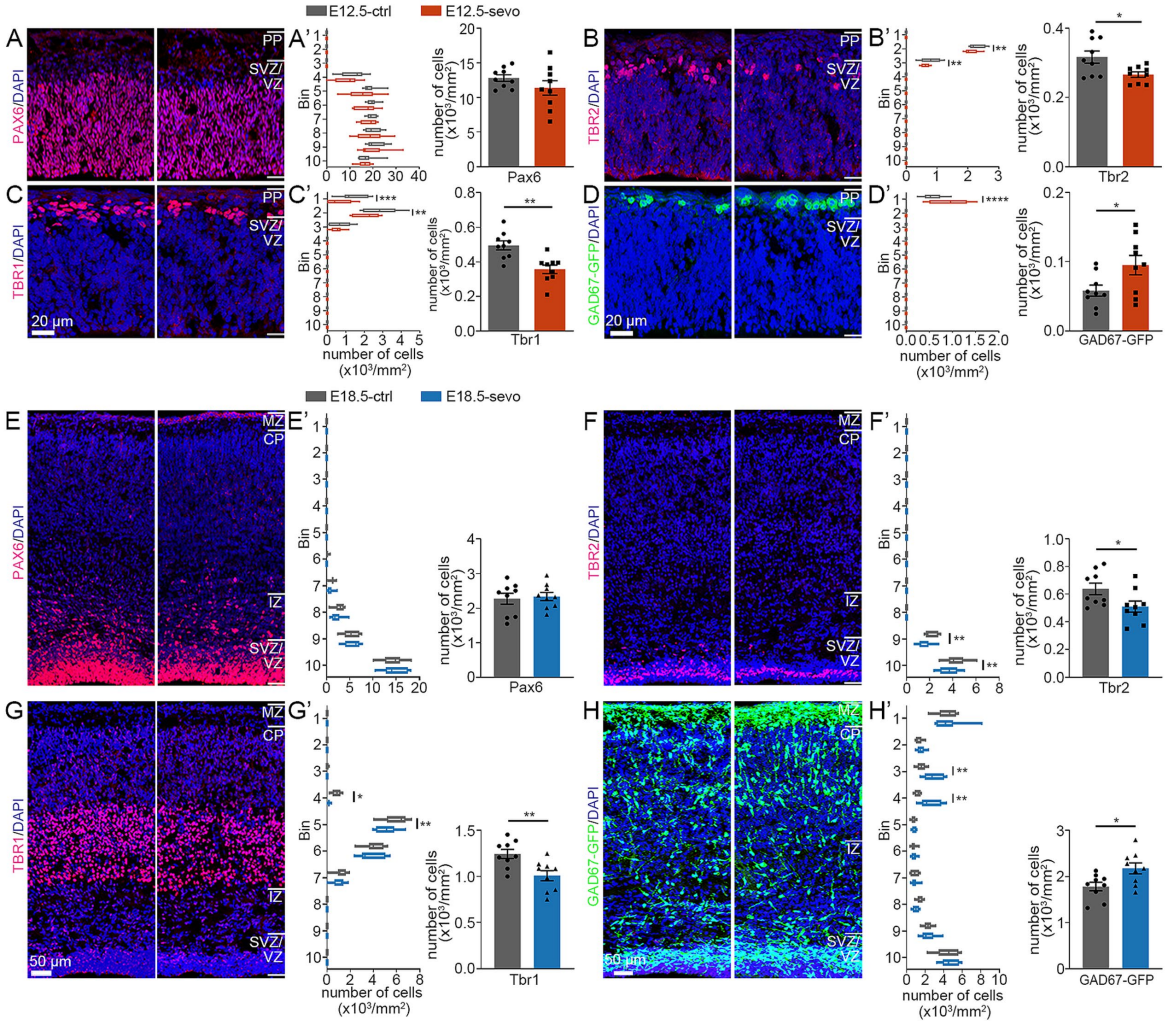
Effects of PSE on cortical Pax6^+^, Tbr2^+^, Tbr1^+^ and GAD67^+^ cells within the E12.5 and E18.5 mouse cortices. **(A–D)** Coronal sections of E12.5 brains of wild-type mice **(A–C)** and GAD67-GFP mice **(D)** treated with air or sevoflurane and evaluated for the presence of Pax6^+^ cells **(A)**, Tbr2^+^ cells **(B)**, Tbr1^+^ cells **(C)** or GFP^+^ cells **(D)** in a standard section of the dorsomedial cerebral wall using a grid divided into 10 bins as described in the text. Pax6^+^ cortical cells were not significantly affected by sevoflurane treatment **(A,A’)**. The distribution of Tbr2^+^ and Tbr1^+^ cells was reduced following sevoflurane treatment **(B,B’,C,C’)**, whereas the distribution of GFP-expressing GAD67^+^ cells at E12.5 **(D,D’)** increased. **(E,F)** Coronal sections of E18.5 brains of wild-type mice **(E–G)** and GAD67–GFP mice **(H)** treated with air or sevoflurane and evaluated for the presence of Pax6^+^ cells **(E)**, Tbr2^+^ cells **(F)**, Tbr1^+^ cells **(G)** or GFP^+^ cells **(H)** in a standard section of the dorsomedial cerebral wall using a grid divided into 10 bins as described in the text. Pax6^+^ cortical cells were not significantly affected by sevoflurane treatment **(E,E’)**. The distribution of Tbr2^+^ and Tbr1^+^ cells was reduced following sevoflurane treatment **(F,F’,G,G’)**, whereas the distribution of GFP-expressing GAD67^+^ cells at E18.5 **(H,H’)** increased. *n* = 9 mice/group. Two-way ANOVAs with Bonferroni *post hoc* correction or two-tailed *t* test were performed for **(A’–H’)**; *p < 0.05; **p < 0.01; ****p < 0.0001.

No significant differences in the distribution of Pax6^+^ neurons in the SVZ/VZ were noted between the E12.5-sevo group and the control group (Figures 2A,A’). The percentage of Tbr2^+^ neurons in Bin 2 and 3 was significantly lower than that in the control group (Figures 2B,B’). The percentage of Tbr1^+^ neurons in Bins 1 and 2 was significantly lower than that in the control group (Figures 2C,C’). The percentage of GAD67^+^ neurons in Bin 1 was significantly greater than that in the control group (Figures 2D,D’). No significant differences in the distribution of Pax6^+^ neurons in the SVZ/VZ were noted between the E18.5-sevo group and the control group (Figures 2E,E’). The percentage of Tbr2^+^ neurons in Bins 9 and 10 was significantly lower than that in the control group (Figures 2F,F’). The percentage of Tbr1^+^ neurons in Bins 4 and 5 was significantly lower than that in the control group (Figures 2G,G’). The percentage of GAD67^+^ neurons in Bins 3, 4 and 10 was significantly greater than that in the control group (Figures 2H,H’). These data show that PSE at either E12.5 or E18.5 suppresses the radial migration of glutamatergic neurons and promotes the tangential migration of GABAergic neurons, with similar effects on embryonic neuronal migration regardless of exposure to sevoflurane at E12.5 or E18.5.

### PSE at different cortical periods influences the laminar distribution of glutamatergic neurons and GABAergic neurons at postnatal day 0 (P0)

**Figure 3:**
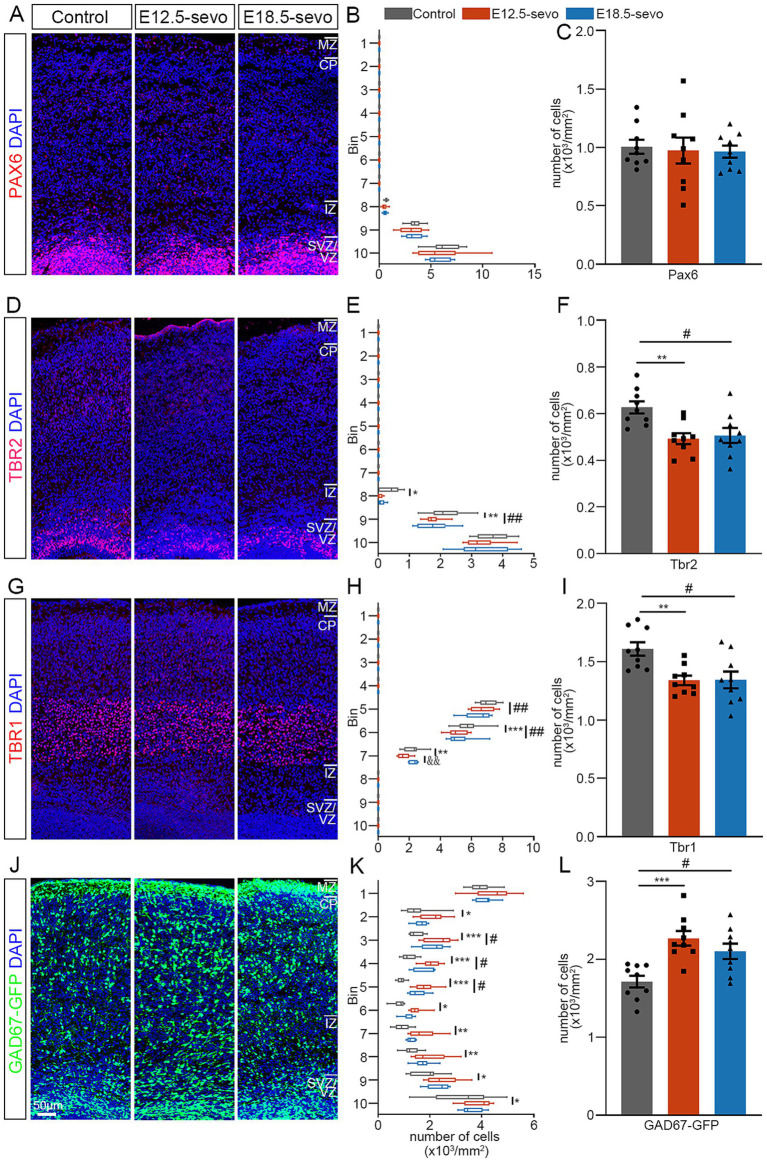
Effects of PSE on cortical Pax6^+^, Tbr2^+^, Tbr1^+^ and GAD67^+^ cells within the P0 mouse cortex. **(A–L)** Coronal sections of P0 brains of wild-type mice **(A–I)** and GAD67-GFP mice **(J–L)** treated with air or sevoflurane and evaluated for the presence of Pax6^+^ cells **(A)**, Tbr2^+^ cells **(D)**, Tbr1^+^ cells **(G)** or GFP^+^ cells **(J)** in a standard section of the dorsomedial cerebral wall using a grid divided into 10 bins as described in the text. Pax6^+^ cortical cells were not significantly affected by sevoflurane treatment **(A–C)**. The distribution of Tbr2^+^ and Tbr1^+^ cells decreased following sevoflurane treatment **(D–F,G–I)**, whereas the distribution of GFP-expressing GAD67^+^ cells at P0 increased **(J–L)**. *n* = 9 mice/group. Two-way ANOVA with Bonferroni post hoc correction was performed for **(B,E,H,K)**, and two-tailed *t*-tests were performed for **(C,F,I,L)**; control vs. E12.5-sevo, *p < 0.05; **p < 0.01; ***p < 0.001. Control vs. E18.5-sevo, ^#^*p* < 0.05; ^##^*p* < 0.01. E12.5-sevo vs. E18.5-sevo, ^&&^*p* < 0.01.

At P0, lamination of the cerebral cortex, which contains six typical layers, has already formed. Therefore, to further evaluate whether different durations of PSE result in differences in persistent abnormalities in the cerebral cortex, we compared the laminar distributions of Pax6^+^, Tbr1^+^, Tbr2^+^ and GFP^+^ neurons in P0 brains after PSE at E12.5 and E18.5. PSE has persistent effects on the forebrain at different developmental stages. These effects include the number or radial migration of Pax6-expressing cells (Figures 3A–C), a decrease in the number of Tbr2^+^ cells and Tbr1^+^ cells in P0 offspring mice (Figures 3D–I), and the inhibition of the radial migration of Tbr2^+^ cells and Tbr1^+^ cells (Figures 3E,H) while promoting the tangential migration of GABAergic neurons (Figures 3J–L). Interestingly, we found that the intergroup differences in the distribution of Tbr1^+^ cells in the PFC of P0 offspring in the two PSE groups were related to the embryonic developmental stage at which exposure to sevoflurane occurred. Exposure during the early stages of cortical development leads to a greater reduction in Tbr1^+^ cells in the ventral cortex. Conversely, exposure during the later stages of cortical development results in a more significant reduction in the distribution of Tbr1^+^ cells in the dorsal cortex (Figure 3H).

### PSE at different cortical periods results in long-term abnormalities in GABAergic and glutamatergic neuron distributions in the cortex

**Figure 4:**
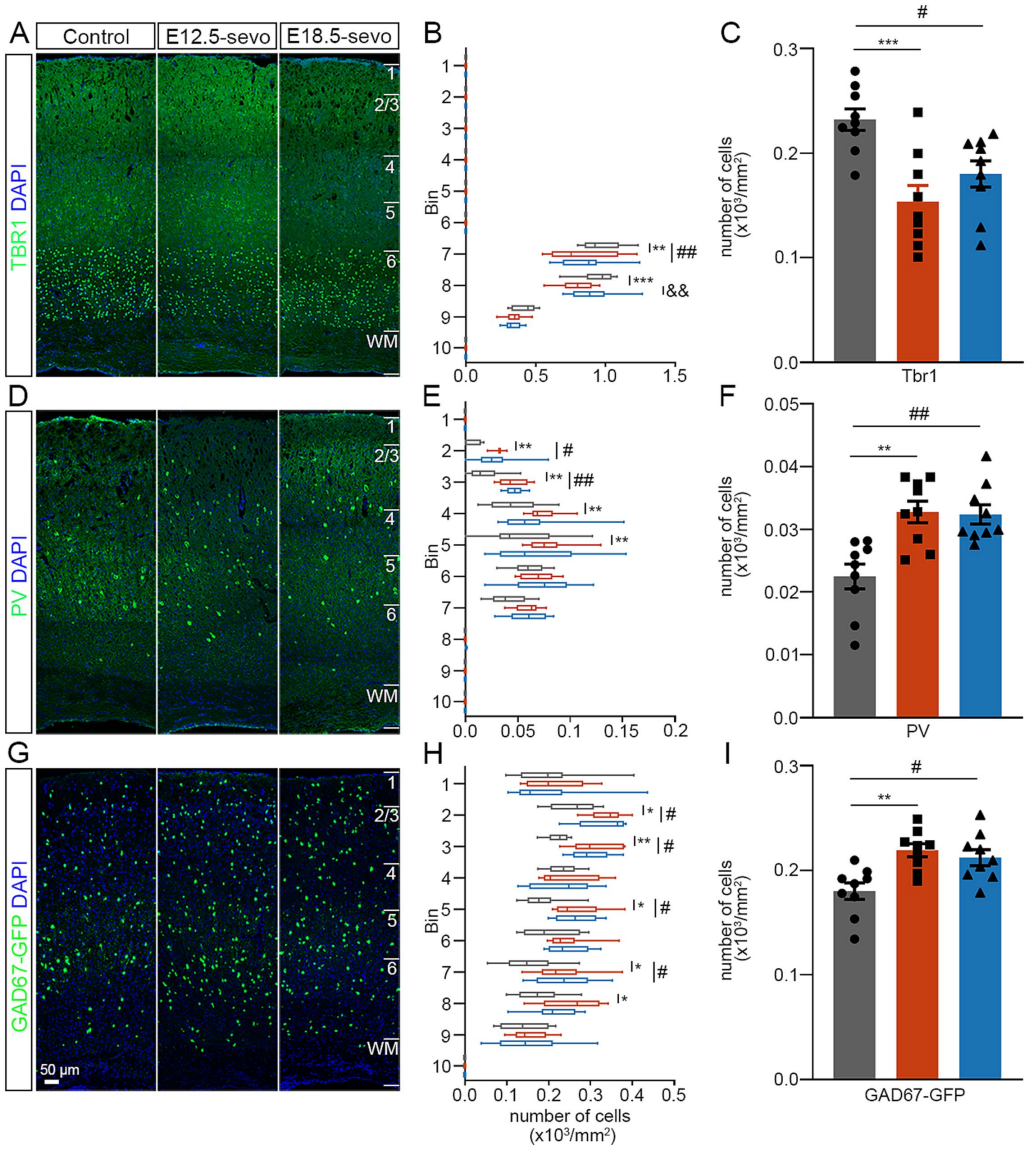
Effects of PSE on cortical Tbr1^+^, PV^+^, and GAD67^+^ cells within the P30 mouse cortex. **(A–I)** Coronal sections of P30 brains of wild-type mice **(A,D)** and GAD67-GFP mice **(G)** treated with air or sevoflurane and assessed for the presence of Tbr1^+^ cells **(A)**, PV^+^ cells **(D)** or GFP^+^ cells **(G)** in a standard section of the dorsomedial cerebral wall using a grid divided into 10 bins as described in the text. The distribution of Tbr1^+^ cells decreased following sevoflurane treatment **(A–F)**, whereas the distribution of PV^+^ cells and GFP-expressing GAD67^+^ cells at P30 increased **(G–I)**. *n* = 9 mice/group. Two-way ANOVA with Bonferroni post hoc correction was performed for **(B,E,H)**, and two-tailed *t* tests were performed for **(C,F,I)**; Control vs. E12.5-sevo, *p < 0.05; **p < 0.01; ***p < 0.001. Control vs. E18.5-sevo, ^#^*p* < 0.05; ^##^*p* < 0.01. E12.5-sevo vs. E18.5-sevo, ^&&^*p* < 0.01.

To evaluate whether these early migration defects impact the mature cerebral cortex, we analyzed the distribution of GABAergic and glutamatergic neurons in the P30 brain. To test these effects, we prepared brain sections from P30 and performed immunofluorescence staining as follows: Tbr1, a marker for glutamatergic neurons (Mihalas and Hevner, 2017); PV, a marker for GABAergic interneurons (Tremblay et al., 2016); and GAD67, a marker for GABAergic interneurons in the neocortex (Balázs et al., 2017). Our results revealed that the density of Tbr1^+^ neurons was significantly decreased in the PSE groups (Figures 4A–C), and PV^+^ and GAD67^+^ neurons are significantly increased in the PSE groups compared to the control (Figures 4D–I). These data suggest that exposure to sevoflurane at both E12.5 and E18.5 has detrimental effects on the divergent plasticity in GABA/glutamate systems created an imbalanced relative numbers of excitatory and inhibitory neurons that progressively impaired network stability, leading to the cumulative effects on the later network dynamics of the brain. Interestingly, similar to the findings observed during the P0 period, early exposure at E12.5 resulted in a greater reduction in cortical ventral Tbr1^+^ cells compared to exposure at E18.5.

### PSE at different cortical periods results in pyramidal neuron atrophy in offspring at the young adult stage

**Figure 5:**
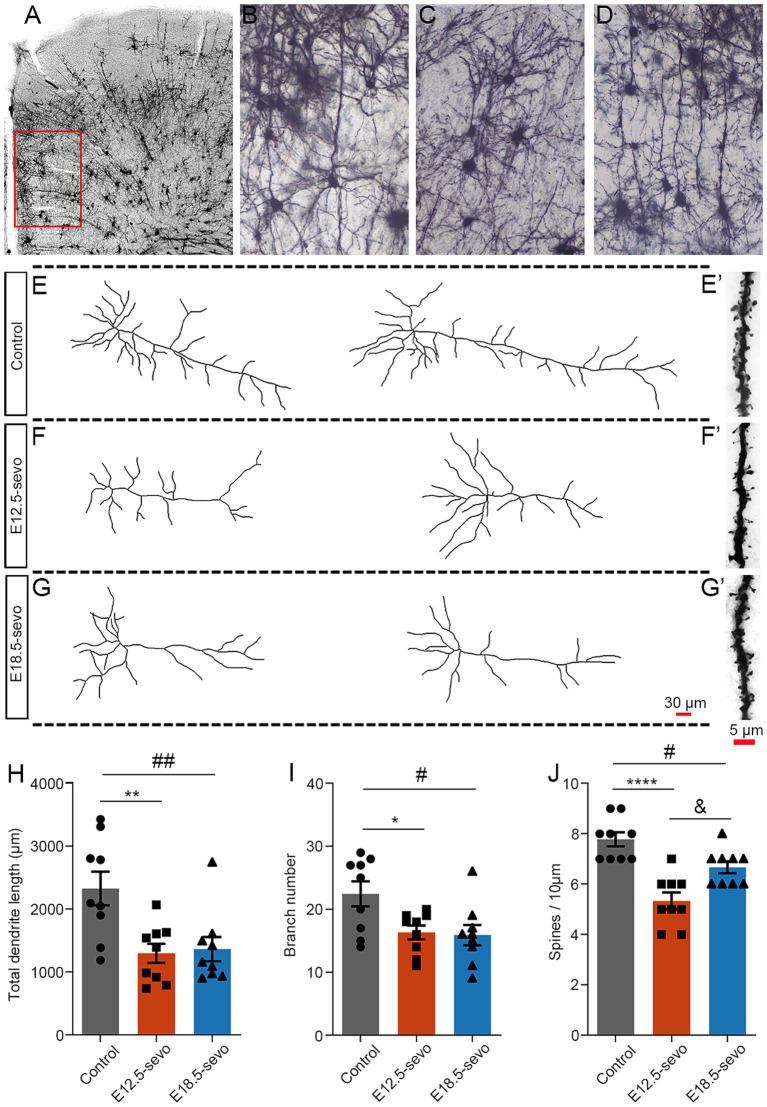
Golgi staining revealed pyramidal neuron atrophy in the mPFC at P30 after PSE. **(A–D)** Golgi-Cox impregnation revealed the morphological structures of pyramidal neurons in laminae II/III of the mPFC at P30 in the control **(B)**, E12.5-sevo **(C)** and E18.5-sevo **(D)** groups. Stained neurons were selected for analysis, as indicated in the boxed area **(A)**, at different postnatal stages. **(E–G,E’–G′)** Examples of reconstructed Golgi-impregnated pyramidal neurons in the P30 mPFC in the control group **(E)** and the PSE **(F,G)** at E12.5-sevo and E18.5-sevo, respectively. Representative spines of basal dendrites in the control **(E’)** and PSE **(F’,G’)** groups at E12.5-sevo and E18.5-sevo, respectively. **(H–J)** Statistics showing a significant decrease in total dendritic length **(H)**, dendritic branch number **(I)** and spine density **(J)** at E12.5-sevo and E18.5-sevo. *n* = 9 mice/group. One-way ANOVA with Bonferroni post hoc correction was performed for **(H–J)**; Control vs. E12.5-sevo, *p < 0.05; **p < 0.01; ****p < 0.0001. Control vs. E18.5-sevo, ^#^*p* < 0.05; ^##^*p* < 0.01. E12.5-sevo vs. E18.5-sevo, ^&^*p* < 0.05.

As described above, PSE resulted in the abnormal distribution of GABAergic and glutamatergic neurons in the cortex. This abnormality affects pyramidal neuron maturation in the brains of P30 offspring. To address this issue, we performed Golgi staining in P30 brains with PSE at E12.5 and E18.5 and analyzed the morphology of pyramidal neurons in the mPFC (Figure 5A), an important brain region closely associated with many CNS disorders. Basal dendrites are defined as dendrites that extend from the basal pole of the soma of pyramidal neurons, in contrast to apical dendrites, which extend from the apical pole (Uylings and van Pelt, 2002). In the PSE, the total length of basal dendrites was significantly shorter at P30 compared with the control (Figures 5E–G,H). The total basal branch number was significantly lower in the PSE treatment than in the control at P30 (Figures 5E–G,I). However, the total length and branch number of the apical dendrites were comparable between the two groups (data not shown). At P30 (Figures 5E’,F’,G’), the spine density was significantly lower in the PSE group compared to the control group (Figure 5J). Interestingly, we found that exposure at early gestational stage E12.5 had a greater effect on neuronal dendritic spines compared to exposure at later gestational stage E18.5 (Figure 5J).

### Offspring of mice subjected to different cortical stages of PSE exhibit abnormal neurobehaviors

**Figure 6:**
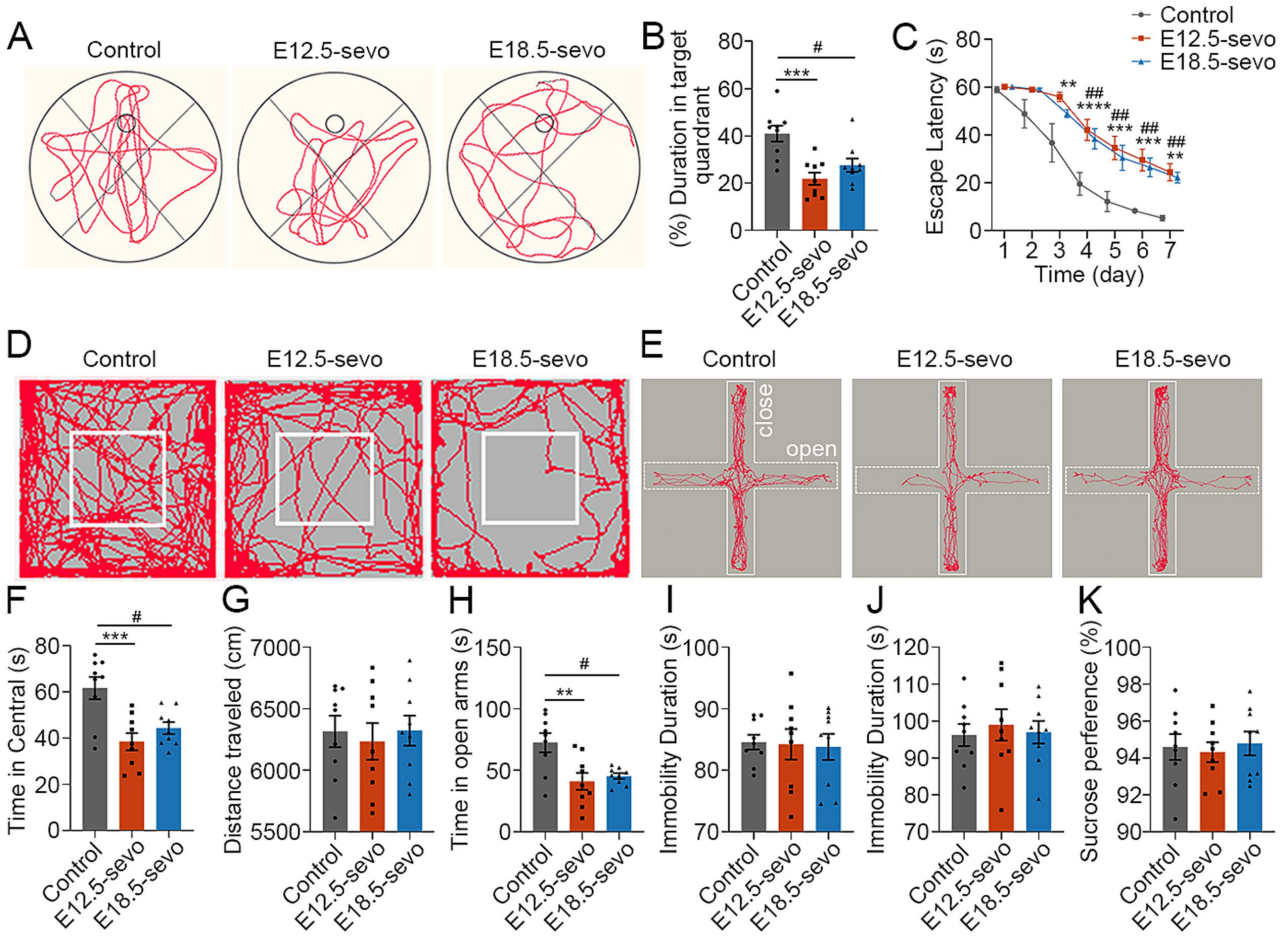
PSE results in anxiety-like behaviors and recognition deficits. **(A–C)** At P30, the MWM test revealed decreased crossings of the platform area **(B)** and increased latency **(C)** to find the platform in the E12.5-sevo and E18.5-sevo groups compared with the control group **(A)**. **(D–H)** At P30, the OFT revealed significantly fewer crossings in the central area within 10 min **(F)** in the E12.5-sevo and E18.5-sevo PSE groups compared with the control **(D)**, but there were no differences in walking distance **(G)** among the three groups. The EPM test revealed significantly fewer crossings in the open arms within 5 min **(H)** in the E12.5-sevo and E18.5-sevo PSE groups compared the control group **(E)**. **(I–K)** At P30, statistical analysis revealed no differences in the TST, FST or SPT among the three groups. *n* = 9 mice/group. Two-way ANOVA with Bonferroni post hoc correction was performed for **(C)**, and one-way ANOVA with Bonferroni post hoc correction was performed for **(B,F,G–K)**; Control vs. E12.5-sevo, **p < 0.01; ***p < 0.001; ****p < 0.0001. Control vs. E18.5-sevo, ^#^*p* < 0.05; ^##^*p* < 0.01.

The E/I balance, which is crucial for brain functions such as emotion, learning, and memory, relies on the relative numbers and maturation of cortical neurons (Pollock et al., 2014; Legon et al., 2016; Selten et al., 2018). We assessed neurobehaviors in P30 offspring subjected to PSE at different cortical stages, using the OFT, EPM, TST, FST, SPT and MWM. Compared with the control groups, the PSE groups spent less time in the target quadrant (Figures 6A,B) and took longer to find the platform (Figure 6C). In the OFT, the central area crossing frequency decreased significantly in the PSE groups (Figures 6D,F), although the total 10-min moving distance was unchanged (Figure 6G). The EPM apparatus is composed of two open arms and two enclosed arms, with the center representing the intersection of these four arms. Time spent in the center is excluded from the total time spent in either the open or enclosed arms. The results of the EPM test demonstrated a reduction in open-arm crossings in the PSE groups (Figures 6E,H). No significant differences were detected in the TST, FST, or SPT results among the groups (Figures 6I–K). These data suggest that PSE at E12.5 and E18.5 causes significant memory deficits and anxiety-like behavior, with no significant behavioral differences observed between early and late exposure.

## Discussion

This study assessed how sevoflurane exposure during different embryonic stages affects offspring, with a focus on neuron development, migration, pyramidal neuron atrophy, and emotional and cognitive abilities. Our data indicate that PSE increases the risk of anxiety and cognitive deficits. This neurotoxicity is linked to the disruption of PFC neural progenitor cell differentiation and migration by sevoflurane, resulting in abnormal glutamatergic and GABAergic neuron development. Moreover, early exposure to sevoflurane may cause greater damage to the fetal forebrain.

Neurotransmitter regulation and environmental exposure during embryonic development constitute critical determinants of neural development and behavioral outcomes. Emerging evidence indicates that early embryonic expression of GABA and glutamate plays a pivotal role in orchestrating electrical activity to establish neurotransmitter specification (Root et al., 2008). Furthermore, environmental perturbations during embryogenesis exert profound effects on neurotransmitter switching in the neonatal mouse cortex, and such transmitter phenotype alterations may predispose individuals to cortical circuit deficits and behavioral abnormalities in adulthood (Godavarthi et al., 2024). As a dual-action agent that acts as a GABA_A_ receptor agonist and NMDA receptor antagonist, sevoflurane exposure represents a unique environmental risk factor. Neurodevelopmental interference likely occurs through two synergistic mechanisms: potentiating GABAergic signaling (via GABAA receptor hyperactivation) and suppressing glutamatergic transmission (via NMDA receptor blockade). These dual disruptions may induce neuronal mispositioning by dysregulating electrophysiological patterns essential for migration (Jevtovic-Todorovic et al., 2013; Gascoigne et al., 2022), thereby compromising overall neural circuit assembly. Of particular developmental importance is the trophic role of ambient glutamate and GABA within neurogenic niches. Unlike their canonical neurotransmitter functions in the mature brain, these molecules primarily mediate neurodevelopmental processes during embryogenesis (Ben-Ari et al., 2007; Ruediger and Bolz, 2007). Experimental studies have demonstrated their regulatory influence on neuronal proliferation, fate determination, and differentiation, with GABA itself functioning as a migratory termination signal (Ben-Ari et al., 2007; Ruediger and Bolz, 2007; Yuan, 2008). Pharmacological agents targeting these systems during pregnancy—including ethanol and antiepileptic drugs—induce persistent neurodevelopmental impairments through mechanisms involving disrupted neurogenesis and aberrant neuronal migration (Berman and Hannigan, 2000; Cuzon et al., 2008). Our findings reveal that embryonic sevoflurane exposure leads to abnormal cortical distributions of glutamatergic and GABAergic neurons alongside emotional–cognitive deficits in juvenile mice. We propose that sevoflurane-induced neurotransmitter switching may represent a key mechanism underlying these emotional deficits, warranting further investigation into the precise relationship between anesthetic exposure and transmitter phenotype specification during critical developmental windows.

Exposure to surgery and anesthesia can occur at different stages of pregnancy. To investigate whether the impact on mouse forebrain development varies with the stage of embryonic exposure to sevoflurane, we selected two critical time points in embryonic brain development (E12.5 and E18.5). Sevoflurane can activate GABA receptors, which play crucial roles in cortical neuron development, with GABA_B1_ and GABA_A-α3_ subunits widely expressed during early embryogenesis (Laurie et al., 1992). These receptors are involved in neural progenitor cell proliferation, migration, and differentiation (Luján et al., 2005). Glutamatergic neurons and their progenitors express Pax6 (Laurie et al., 1992; Luján et al., 2005), whereas GABAergic interneurons express Mash1 in their proliferative zones (Letinic et al., 2002). Our *in situ* hybridization experiments revealed that at two key embryonic stages—early neurogenesis (E12.5) and late prenatal neuronal migration (E18.5)—Pax6 is expressed in the VZ/SVZ of the PFC and coexists with GABA_B1_ and GABA_A-α3_ receptors, whereas Mash1 is expressed in the GE and coexists with these receptors. GABA signaling has a strong regulatory effect on both radial migration and tangential migration. Research has shown that functional ionotropic GABA_A_ receptors exist in cells involved in radial migration and serve as a stop signal for developing cortical radial migration neurons (Owens et al., 1996). GABA_A_ receptors are involved in the migration of neurons from the proliferative zone into the intermediate zone, whereas GABA_B_ receptors provide signals that prompt cells to migrate into the cortical plate (Heck et al., 2007). Metabotropic GABA_B_ receptors, on the other hand, are reportedly associated with the tangential migration of neurons (López-Bendito et al., 2003). These findings suggest that PSE during different stages of embryo development may differentially affect neuronal migration by influencing GABA receptors.

Although prior studies have demonstrated that 4-h exposure to 2.5% sevoflurane induces neuronal apoptosis in neonatal mice (Zhao S. et al. 2020), the developmental stage-specific impacts on neuronal migration and behavioural outcomes remain unexplored. Notably, extended exposure to 2.5% sevoflurane for 6 h has been shown to elicit cortical maldevelopment and cognitive dysfunction (Song et al., 2017). Our preliminary experiments revealed that 4-h exposure during the E12.5 stage (a critical window for cortical neuronal migration in mice) significantly delays migratory progression and is correlated with marked cognitive deficits and anxiety-like behaviors in juvenile stage. While our study did not directly assess myelination status or apoptosis following 4 h PSE exposure, the 6 h sevoflurane exposure duration has been specifically associated with both myelination suppression (Zuo et al., 2020), and increased inflammation signaling pathways activation (Zhang et al., 2024), potentially confounding phenotypic interpretation through dual mechanisms. Our selection of the 4 h paradigm was therefore designed to capture primary migratory impairments while avoiding the established neurotoxic thresholds reported in these longer-duration exposures. The divergent phenotypic patterns observed between exposure durations—particularly for PAX6^+^ neuronal distribution—hint at temporally stratified mechanisms (Englund et al., 2005).

Ionotropic GABA_A_ receptors themselves act as depolarizing signals to stop the migration of developing cortical radial glial neurons during embryonic development (Furukawa et al., 2014). Our results indicate that sevoflurane exposure led to a reduction in Tbr1^+^ distribution in both PSE groups (Figures 3H, 4B), with notable inhibition of radial migration. Therefore, the inhibition of radial migration by sevoflurane may be due to its activation of GABA_A_ receptors. Interestingly, in the P0 mouse cortex, compared with that in the control group, the decrease in Tbr1^+^ density during early embryonic cortex development (E12.5-sevo group) occurred mainly in the ventral region of the cortical CP area (6–7 bins). In contrast, during late embryonic cortex development (E18.5 exposure group), the decrease occurred mainly in the dorsal region of the CP area (5–6 bins). This may be related to the temporal dynamics of cortical development, during early embryonic development, immature neurons exhibit depolarization upon GABA_A_ receptor activation due to NKCC1-mediated high intracellular chloride concentrations, promoting neuronal migration through chloride efflux (Dzhala et al., 2005; Ben-Ari et al., 2007). However, excessive enhancement of GABA_A_ activity by sevoflurane may disrupt migratory rhythms through chloride depletion or network hyperexcitability triggered by prolonged inhibitory signaling. In contrast, at later developmental stages, the upregulation of KCC2 reduces intracellular chloride levels, converting GABA_A_ receptor activation to hyperpolarization, thereby inhibiting migration (Dzhala et al., 2005; Ben-Ari et al., 2007). Although migratory activity is reduced during this phase, GABA_B_ receptors further modulate the process by suppressing glutamatergic signaling (Sanchez-Vives et al., 2021), which may provide negative feedback regulation effects. Furthermore, the dynamic regulatory compensation between NKCC1 and KCC2 creates a homeostatic balance across developmental stages. This biphasic regulatory mechanism with built-in compensatory pathways ultimately results in limited differential impacts of sevoflurane exposure between early and late developmental periods. We propose that this mechanism likely underlies the observation that early versus late treatment groups demonstrate only minor intensity differences in a few measured parameters rather than substantial overall divergence.

GABAergic interneurons are generated in the GE proliferation zone and migrate tangentially to the dorsal cortex. Our results indicate that after PSE at different stages of cortical development, the number of GAD67-GFP^+^ neurons migrating tangentially to the dorsal cortex increased. These findings suggest that PSE at various stages promotes the tangential migration of inhibitory neurons. This finding is similar to studies on the effects of prenatal alcohol exposure on brain development, where GABA receptors, targeted by both anesthetics and alcohol, are disrupted during pregnancy, which accelerates the tangential migration of fetal cortical GABAergic interneurons (Cuzon et al., 2008; Tochitani et al., 2010; Skorput et al., 2015). Metabotropic GABA_B_ receptors play crucial roles in the tangential migration of cortical interneurons (Luhmann et al., 2015), and blocking these receptors alters the distribution of tangentially migrating interneurons in the cortex (López-Bendito et al., 2002). GABA regulates the migration of embryonic cortical neurons, including GABAergic interneurons, through GABA_A_ receptors (Cuzon et al., 2006). Our results indicate that at different stages of cortical development post-PSE, there is an increased number of GAD67-GFP^+^ tangentially migrating neurons to the posterior cortex. This effect is potentially attributed to changes in GABA-mediated neurotransmission induced by sevoflurane exposure, thereby promoting the tangential migration of GABAergic interneurons. Current studies have not excluded other possibilities, such as whether sevoflurane exposure affects the proliferation of progenitor cells originating from the ganglionic eminence (GE). Research suggests that in humans, although many cortical interneurons originate within the cortex, approximately 35% are generated in subcortical regions and migrate tangentially to the cortex (Letinic et al., 2002). Therefore, this abnormal tangential migration may lead to neurofunctional disorders following sevoflurane exposure.

The offspring were reared until P30, and the immunofluorescence results revealed a selective increase in the number of PV interneurons in the PFC of P30 offspring from the PSE group compared with those from the control group, possibly reflecting increased translocation in early postnatal life. Additionally, the density of Tbr1^+^ cells in the PFC of P30 offspring from the sevoflurane-exposed group was significantly lower than that in the PFC of the control group, indicating the persistent impact of sevoflurane exposure at different stages of embryonic development on the reduced number of Tbr1^+^ cells. However, the differential distribution of Tbr1^+^ cells in the frontal cortex of P0 offspring mice was not observed in P30 offspring mice, likely due to notable positional shifts in Tbr1^+^ expression during mouse brain development (Bulfone et al., 1995). Further experimental investigations are needed to elucidate the underlying reasons. Consequently, the decrease in the number of Tbr1^+^ cells in juvenile mice may contribute to the abnormal distribution and expression of excitatory and inhibitory neurons in the brain, potentially leading to anxiety and cognitive impairments.

Compared with E18.5 exposure, E12.5 exposure induced significantly greater reductions in Tbr1^+^ excitatory neurons within the P30 mouse cortex (Figures 3, 4). As Tbr1 serves as a lineage-specific marker for excitatory cortical neurons (Chung et al., 2024), its diminished expression suggests compromised development of this neuronal population. Previous work has shown that Tbr1^+^ neuron deficits directly impair cortical excitatory drive (Xing et al., 2021), whereas our prior findings demonstrated concomitant increases in inhibitory interneurons postexposure. This dual perturbation synergistically reduces the cortical E/I balance. Notably, the E/I imbalance in the E12.5-sevo group was more severe than that in the control group due to greater excitatory neuron loss, implying enhanced inhibitory dominance over mPFC pyramidal neurons (Figure 4). Mechanistically, studies have demonstrated that a decrease in the E/I ratio is correlated with dendritic spine elimination (Peça et al., 2011). Chronic hyperinhibition may disrupt pyramidal neuron maturation, resulting in simplified dendritic arborization and reduced spine density (Zhao T. et al., 2020). In line with this, golgi staining was performed on pyramidal neurons in the mPFC of P30 offspring, indicating that the PSE-induced E/I inbalance in the PFC may adversely affect the development and maturation of pyramidal neurons, resulting in reduced branching, shorter dendrites, lower spine density, and neuronal atrophy. We therefore propose that exacerbated spine loss following early gestational exposure involves a two-hit mechanism: (I) sevoflurane-induced depletion of excitatory progenitors during the E12.5 neurogenic surge creates a foundational deficit, and (II) the subsequent relative numbers of excitatory and inhibitory neurons imbalance amplifies spine pruning through activity-dependent plasticity cascades. This temporal vulnerability aligns with the heightened sensitivity of early corticogenesis to developmental insults.

To assess the impact of PSE on learning and memory in offspring at different cortical development stages, we conducted the MWM test (Vorhees and Williams, 2006). The results revealed a significant increase in escape latency and reduced time spent in the target quadrant in the PSE group, indicating impaired spatial learning and memory in offspring mice after PSE. Dysregulation of prefrontal cortical neurogenesis, such as incomplete aggregation and an abundance of newborn neurons, may lead to neurological disorders during brain development, as the downregulation of Tbr1^+^ neurons can result in neurological disorders (Notwell et al., 2016). We observed low exploratory behavior patterns in the PSE group, suggesting anxiety-like behavior in offspring mice prenatally exposed to sevoflurane. This effect was potentially attributed to abnormal differentiation and migration caused by PSE, resulting in an uneven distribution of excitatory and inhibitory neurons, ultimately disrupting the E/I balance. Similar studies have shown (Fazel Darbandi et al., 2018) that a decrease in Tbr1^+^ cells in the mouse PFC can also affect the number of excitatory and inhibitory synapses, disrupting the E/I balance and leading to anxiety-like behavior in mice.

Both our study and prior research examine the neurodevelopmental consequences of PSE, with key differences emerging in exposure timing and underlying mechanisms. Previous studies primarily focused on mid-gestational exposure, identifying mechanisms such as suppression of PFC proliferation/differentiation (Song et al., 2017), impaired neural stem cell specification (Zhang et al., 2022), disrupted interneuron migration (Liang et al., 2023), and postnatal hippocampal myelination deficits (Fan et al., 2022), all contributing to anxiety-like behaviors or cognitive-motor impairments. In contrast, our findings demonstrate that both early-and late-gestational sevoflurane exposure lead to developmental imbalances in glutamatergic/GABAergic systems, with early exposure causing more severe forebrain damage. This extends the vulnerable period to both early and late gestation and introduces a novel mechanism of synaptic circuit imbalance, distinct from prior reports. Specifically, we propose that synchronized disruption of E/I systems, rather than isolated neurogenesis or myelination defects, underpins emotional and cognitive dysfunction. Additionally, our identification of stage-specific vulnerability patterns, such as heightened forebrain susceptibility during early exposure, offers clinically actionable insights for refining anesthesia risk assessments across pregnancy trimesters.

The present study has several limitations that should be considered. First, we did not determine the long-term effects of sevoflurane anesthesia on learning and memory function, however, the current findings were able to illustrate the effects of sevoflurane anesthesia on behavioral changes (e.g., spatial learning and memory impairment). Second, we only focused on 2.5% sevoflurane concentration and a 4-h exposure period. Different concentrations and exposure durations may exert distinct effects on fetal development because of the neurotoxicity caused by anesthetic exposure is primarily dependent on the concentration of the anesthetic and the duration of exposure. Future research to investigate the effects of different concentrations and exposure durations on fetal development is necessary. Thirdly, it has been suggested that translating the results of preclinical studies that involve the mouse model to humans might be misleading. However, it is obvious that ethical considerations create a major limitation with regards to carrying out research on human newborns. We firmly believe that preclinical studies involving mice can still yield valuable information relating to the mechanisms underlying neurotoxicity induced by exposure to anesthetics. Finally, while rodent models provide valuable insights into the basic mechanisms of sevoflurane-induced developmental effects, we fully acknowledge the inherent limitations. Key considerations include: (I) Physiological disparities in drug metabolism rates between rodents and humans. (II) Species-specific differences in neural developmental timelines. (III) Potential variability in placental transfer efficiency, which may lead to differential fetal exposure levels under identical maternal dosing regimens. These interspecies distinctions necessitate cautious interpretation of dose–response relationships and temporal exposure thresholds. Future studies incorporating non-human primates or human-derived organoid models would be critical to bridge this translational gap.

In summary, this study extensively analyzed the effects of PSE on embryonic brain cortical development at different stages. These results indicate that PSE alters the differentiation and migration of cortical glutamatergic and GABAergic neurons, leading to learning, memory, and anxiety-like behaviors neurobehavioral dysfunctions in offspring. Notably, early PSE may cause greater damage to the fetal forebrain. These findings enhance our understanding of PSE-induced neurotoxicity and highlight the need to consider the neurotoxic effects and timing of anesthesia during pregnancy when evaluating the benefits and risks of nonobstetric surgery.

## Data Availability

The original contributions presented in the study are included in the article/supplementary material, further inquiries can be directed to the corresponding author/s.
